# Early Mistreatment Contributes to Social Behavior Disorders in Zebrafish

**DOI:** 10.3389/fnbeh.2020.578242

**Published:** 2020-10-16

**Authors:** Fabiano Peres Menezes, Igo Padilha de Sousa, Ana Carolina Luchiari

**Affiliations:** Departamento de Fisiologia e Comportamento, Centro de Biociências, Universidade Federal do Rio Grande do Norte, Natal, Brazil

**Keywords:** *Danio rerio*, stress, aggression, social, juvenile, cohesion

## Abstract

Adverse experiences during childhood have been the focus of a series of studies due to the psychological damage observed in individuals who suffered abuse during their youth. Studies with model animals that can mimic these observations can significantly contribute to understanding the mechanisms behind this phenomenon. In our experiments, young zebrafish (20 dpf) were exposed to aggressive alcoholized male adults for 30 min for 10 days. At 30 dpf, the animals were tested for shoal formation, and at 60 dpf, locomotion and aggression were evaluated. Animals that suffered oppression from adults showed greater group cohesion and lower attack emission and higher distance from the image in the mirror test. Locomotor parameters were not changed. These results show that the stress caused by aggression exposure in the juvenile phase led to increased fear and avoidance behavior later in life. Moreover, we confirm the importance of the zebrafish as a sensitive tool for studies on the effects of early mistreatment and its consequences to adult behavior.

## Introduction

Traumatic experiences during childhood, such as physical and sexual abuse, helplessness, and mistreatment, are worldwide concerns. Several studies show that, although many individuals are resilient, early exposure to trauma can significantly increase the chance of developing a series of lifelong psychiatric disorders (Anda et al., 2006; Khoury et al., 2010). Many of them reported the relationship between trauma caused by childhood abuse and drug use in adulthood (Anda et al., 2006).

Although some studies are dedicated to understanding how stress in the juvenile phase can alter adult behavior in humans (Anda et al., 2006; Heim et al., 2008; Bale et al., 2010; Meewisse et al., 2011; Pechtel and Pizzagalli, 2011), studies using animal models to test the correlation between adverse experiences during early developmental phases and the behavioral consequences in adulthood are less representative (Shams et al., 2018; Aponte and Petrunich-Rutherford, 2019). Most studies approach the consequences of perinatal stress, especially intrauterine stress, and little is known about the stressful events experienced during the childhood or prepubertal period (juvenile phase). According to Romeo and McEwen (2006), there are significant changes in brain structure, connectivity, and function during juvenile development, making this phase extremely sensitive to stress. Indeed, the few studies approaching juvenile stress consequences have pointed to anxiety and stress disorders, depression development, and increased suicide attempts in adulthood (Morgan et al., 2003; Kausch et al., 2006; Weich et al., 2009). Thus, studies focusing on the effects of juvenile stress experiences are of high translational interest. For instance, studies in rodents demonstrate that behavioral responses, such as adulthood anxiety, predominates in animals systematically separated from the mother during the early postnatal period (Wei et al., 2010). This is evidence that the correlation, to a certain degree, can present correspondence between model animals and humans.

However, studies on this topic have preponderantly used rodents as models, and although they have been intensely contributing to the knowledge in the field, the stress response in this model is slightly different from humans. During activation of the hypothalamic-pituitary-adrenal (HPA) axis as a response to stress, corticosterone is the primary hormone released by rodents. However, the cortisol hormone is the one released by humans. Although both hormones are similar in the way of action and both can pass through the blood–brain barrier and alter brain functioning, animal models that discharge cortisol are more reliable for translational research on stress effects. Moreover, disconnecting the stress effects during the intrauterine phase from the stress during the postnatal period in mammals is difficult, and models that allow distinguishing the developmental phases may be of interest in stress studies.

In this sense, the zebrafish is the ideal compromise between the physiological (cortisol release instead of corticosterone) and behavioral responses, and the facility of breeding and manipulation avoids stress interference before the juvenile phase of development. In zebrafish, whose characteristics differ from mammals in terms of parental care, social structure, and types of intraspecific interactions, animals that experience stress in the juvenile phase do not exhibit anxiety-related behaviors in adulthood but show differences related to memory (Paull et al., 2010; Séguret et al., 2019; Fontana et al., 2020). However, new approaches need to be explored to better understand the long-term responses of zebrafish to disturbances in the early stages of development. Therefore, this study’s objective was to evaluate the zebrafish behavioral response after physical and psychological abuse caused by an older and aggressive conspecific during the juvenile phase and to assess the impact of the stressful experience later in life.

## Materials and Methods

The animals used in this work came from the breeding carried out in the FishLab-UFRN. Breeding matrices from the second generation of zebrafish reared at FishLab were used to obtain embryos. The matrices were kept in 7-l tanks in a ratio of 10 males to five females, housed in a continuous flow-reverse osmosis tank shelf with a pH of 7.5 and salinity from 0.04 ppt. The system has a series of probes (pH and conductivity) and filters (mechanical and biological) that control water quality. The light cycle maintained is 12 h light and 12 h dark. The matrices were fed three times a day with flocked ration and one time a day with artemia nauplius. For breeding, males and females were transferred to a breeding tank in a ratio of two males to one female, where an acrylic barrier separated them for 14 h, and at the beginning of the first hour of the light phase, the barrier was removed, and breeding occurred (protocol in agreement with Westerfield, 2007). After spawning, the matrices were returned to the home tanks, and eggs were collected with a Pasteur pipette. All protocols were submitted for approval by the Ethics Committee for Animal Use (CEUA-UFRN), and followed Brazilian law, according to the guide of the National Council for Animal Experimentation Control—CONCEA.

### Animal Groups

After oviposition, 30 eggs were placed in a 30 × 20 cm (width × length) tank with system water at 2 cm height. Larvae started to receive paramecium and powdered food (3× a day) from 6 dpf (days post-fertilization). At 10 dpf, the larvae were divided into three groups of 10 animals each, which formed the groups Naïve (Nv), Control (Ctr), and Oppressed (Op). Groups were allocated in 3.5-l tanks covered in blue (housing tank) and were fed with ground food and brine shrimp. When fish reached 20 dpf, the four smallest animals in each group were removed to keep size homogeneity and prevent aggression from bigger animals. From 20 to 30 dpf, the juvenile fish were exposed to aggressive male adult fish in a controlled condition (described below).

### Aggression Exposure

The selection of the aggressor animals was carried out *via* a clash between the animals, in which a trained observer determined the occurrence of agonistic behavior. Forty adult animals (males, 6 months old, wild type) were separated and distributed in pairs, in 2-l tanks, and observed for a maximum of 30 min regarding chasing behavior. The couples that did not show any persecution behavior during that period were rearranged in new duels. The chasing and attacking animals were considered the winners of the match. The first 12 winners of each pairing went on to a second pairing held only between the winners. Thus, the 6 animals with the highest propensity to aggressive behavior were selected. After selection, the attackers were kept in 7.5-l tanks until the tests with juveniles.

Before the adult aggressor–juvenile pairing, the aggressive adult males were individually exposed to 0.5% ethanol for 1 h to intensify the aggressive behavior (Gerlai et al., 2000; Sterling et al., 2015). Immediately after ethanol exposure, the animals were washed in system water (drug-free), where they remained for 30 s, to eliminate ethanol vestiges. Shortly after cleaning, the pairing procedure took place.

The tank used for adult aggressor–juvenile pairing was a 15 × 15 cm tank filled to a depth of 3 cm. The bottom and three walls of the tank were covered with a black-and-white grid printed on adhesive article. The grid was used to offer an environmental cue to the juvenile’s association with the stressing situation. The animals designated to the Oppressed group (Op) were then individually placed in the grid tank. After 1 min acclimatization, alcoholic aggressive animals were introduced into the tank, thus forming an adult aggressor–juvenile attacked pair. The exposure time lasted 30 min and was performed every day for 10 days. The pair formation was randomly defined each day, so that aggressor fish changed every day and hierarchy establishment was avoided.

The Control group animals (Ctr) spent 30 min in the same tank used for the adult aggressor–juvenile pairing (tank covered in grid article) but without being exposed to aggressive fish. The naïve animals (Nv) remained in the housing tank throughout the experimental period until the test day.

### Cohesion Test

Twenty-four hours after the last aggressor exposure, fish were tested for group cohesion. For that, we used a tank of 30 × 15 × 20 cm filled to a depth of 4 cm. The test tank had 3 walls and the bottom covered in blue, similar to the housing tank, and one of the walls was covered with the same grid used for the aggression exposure.

The animals were gently transferred in groups of 4, all at once, to the testing tank and recorded for 5 min. After that, each group of animals returned to their housing tank. The videos obtained went through a process in which a frame was extracted every 10 s, using Freestudio software, generating 30 images analyzed for the average distance between the individuals in each group, using the ImageJ software.

### Novel Tank and Mirror Test

When the animals from each group (Op, Ctr, and Nv) completed 60 dpf, fish were tested for locomotion and aggression. We used a vertical tank measuring 20 × 20 × 8 cm filled to a depth of 15 cm. One of the side walls was covered with the grid article used before to recover the stressing situation memory.

Each animal was recorded for 10 min, in which locomotion and attack emission were measured. After recording fish behavior for 5 min, a mirror was carefully displayed at the side opposite the grid wall and another 5 min of behavior was recorded.

For the locomotion analysis, the tank was virtually divided into three horizontal areas: the bottom, middle, and top 5 cm, which were considered bottom, middle, and upper zones of the tank and locomotion between and inside each area was evaluated.

The handling time for placing the mirror was discarded from the analysis. The water in the test tank was changed after each test to avoid any interference caused by a possible release of an alarm or other substances.

Any-Maze screening software (Stoelting Co., USA) was used to measure behaviors. The following parameters were considered: latency to enter the upper zone, the average distance from the lower zone, time in the lower zone, average speed while moving, and mobile time. During the mirror test, the average distance maintained from the mirror area and the number of frontal attacks against the mirror were also measured.

### Statistical Analysis

Statistical tests were performed using Graphpad-Prism software version 6.0 (La Jolla, CA, USA). The normality test was performed in all groups through the D’Agostino and Pearson normality test. To check for significant differences between groups, one-way ANOVA was used, followed by Dunnett’s *post hoc* test for multiple comparisons. Two-way ANOVA was used to test differences in mirror distance among the groups, considering the time (before and after mirror) and group (Ctr, Nv, and Op). Statistically significant level was considered *p* < 0.05. Values were expressed as means and standard deviation.

## Results

### Cohesion Test

**Figure 1:**
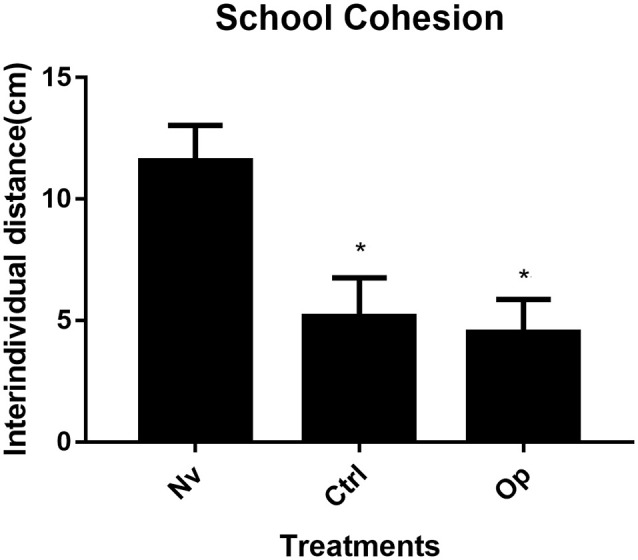
Average interindividual distance (n:3; pool 4) measured from 30 image frames extracted from 5 min behavioral record. Data were expressed as mean ± SD for each group and were analyzed by one-way ANOVA (average distance) followed by Tukey’s *post hoc* test. *Denotes statistical significance from the Naive (Nv) group *p* < 0.05.

The results presented for the cohesion behavior test demonstrate statistical significance between groups (one-way ANOVA *F*_(2,9)_ = 18.82, *p* < 0.05). The control group individuals presented an average interindividual distance of 5.5 cm that was statistically lower than the naïve group (*p* < 0.006). The oppressed group, with an average interindividual distance of 4.51 cm, was statistically lower than the naïve group (*p* < 0.001). The average interindividual distance for the naïve group was 11.57 cm (Figure 1).

### Novel Tank Test

**Figure 2:**
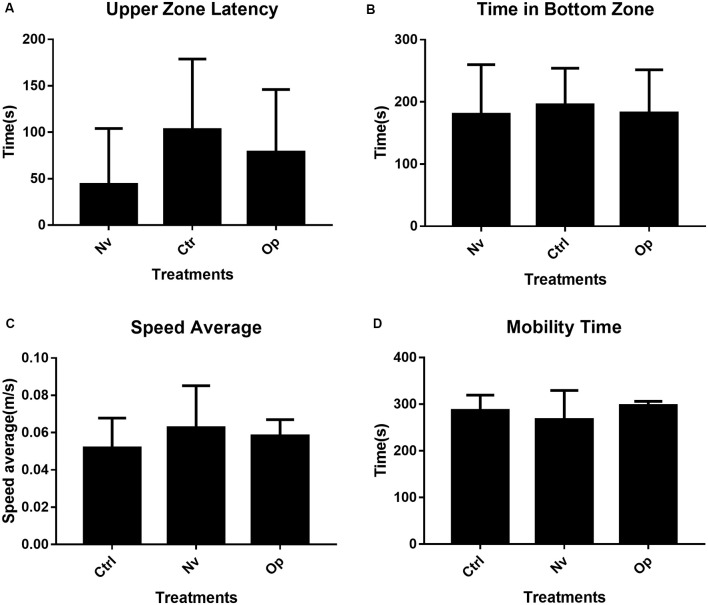
Locomotor activity in novel tank aparatus during 5 min for Naive (Nv), Control (Ctrl), and Oppressed (Op). **(A)** Upper zone latency (s). **(B)** Time in bottom zone (s). **(C)** Speed average during movement (m/s), and **(D)** mobility time. The bars represent the mean ± SD. N:11 represents the number of animals per group. No group shows means statistical significance at *p* < 0.05 in relation to Naive group.

The results extracted from the tests carried out for locomotion evaluation did not show statistical significance. Although the average time for the first climb to the top of the tank is visually different, the statistical tests point to a considerable variation between individuals, thus not expressing statistical significance between groups (*F*_(2,30)_ = 2.05, *p* > 0.05; Figure 2). One-way ANOVA did not show statistical significance for time spent at the bottom of the tank (*F*_(2,30)_ = 1.18, *p* > 0.05), average speed (*F*_(2,30)_ = 1.18, *p* > 0.05), and mobile time (*F*_(2,30)_ = 1.55, *p* > 0.05; Figure 2).

### Mirror Test

**Figure 3:**
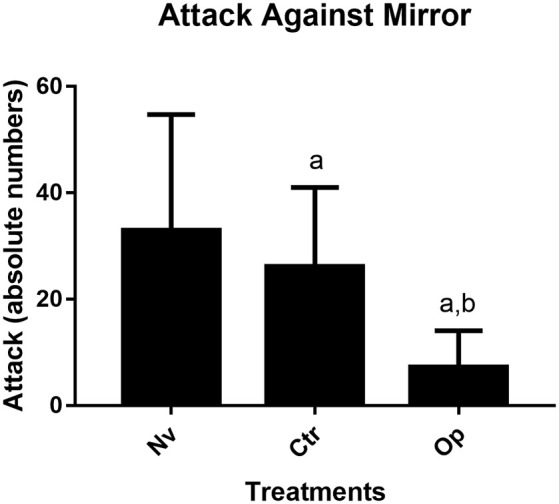
Number of aggressive attacks made againt a mirror image in the mirror test for 5 min recorded test for Naive (Nv), Control (Ctr), and Oppressed (Op) groups. Data were expressed as mean ± SD for each group (n: 10) and were analyzed by one-way ANOVA followed by Tukey’s *post hoc* test. (a) Denotes a significant difference from the Naive (Nv) group *p* < 0.05 and (b) a significant difference from the Control (Ctr) group *p* < 0.05.

To determine the level of aggression, we use the most classic parameter, which consists of direct attacks against the mirror. The results obtained demonstrate that the animals exposed to aggressive adult fish (Op) during the juvenile phase presented a significantly lower average number of attacks against the mirror when compared to both the Naive group (Nv) and the control group (Ctrl; *p* < 0.003 and *p* < 0.04, respectively). The Ctrl and Nv groups did not show statistical differences (*F*_(2,27)_ = 7.21; Figure 3).

**Figure 4:**
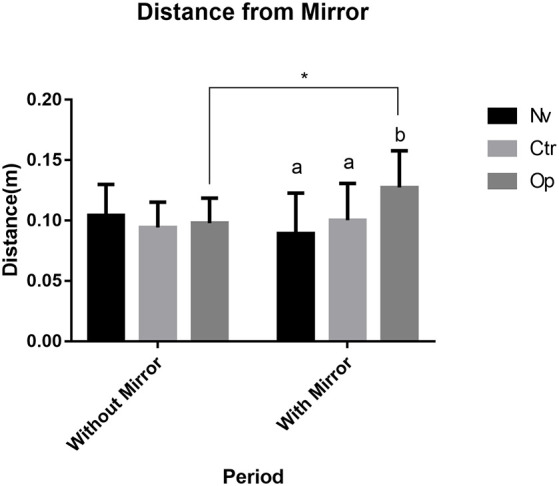
Mean distance for mirror zone before and after the mirror presence during the recorded test for Naive (Nv), Control (Ctr), and Oppressed (Op) groups. Data were expressed as mean ± SD for each group (n: 11). Data were analyzed by two-way ANOVA (average distance) followed by Sidak’s *post hoc* test. Different lower-case letter denotes statistical significance between groups during the mirror presence. *Indicates statistical significance between before and after mirror presence for the Oppressed group.

The average distance for the mirror allowed determining if any of the groups tried to avoid the interaction with a “stranger” projected in the mirror. In our first comparison, we verified the difference between the groups before the presence of the mirror. The results demonstrate that there was no significant difference between the groups before the presence of the mirror (*F*_(2,30)_ = 0.55, *p* > 0.05; Figure 4). However, in the results with the mirror’s presence, the Op group showed a significant difference compared to the Nv group, presenting a greater mean distance from the mirror (*p* < 0.03). In addition, when comparing distance measurements before and after the presence of the mirror within the same group, individuals in the Op group showed an increase in the average distance when in the presence of the mirror (*F*_(1, 30)_ = 1.167, *p* < 0.05; Figure 4).

## Discussion

In this study, we evaluated the effects of the stress caused by several encounters with an aggressive adult fish under the effect of alcohol on zebrafish behavior. Tests were carried out at different stages of development after stress induction. We observed that, shortly after the stress exposure, young zebrafish presented higher group cohesion (Figure 1), and later, it expressed social avoidance (Figure 3) and decreased aggressive behavior (Figure 4). This is the first study to approach the effects of social stress during young development on later animal behavior.

Early-life stress is known to affect neurochemical and behavioral patterns that lead to behavioral issues during adulthood. Some stressors imposed in moderate amounts contribute to increased behavioral flexibility and even improve cognitive performance (Fontana et al., 2020). However, some social stressors, such as the one imposed in this study, may trigger brain alterations that affect the proper behavioral repertoire and lead to psychological deficits. Social stressors have been shown to affect several species and depend on the stressor type, timing, and intensity to impact physiology and behavior. In a social species, such as the zebrafish, shoaling appears during animal development with the animals showing greater cohesion as they approach adulthood (Buske and Gerlai, 2011) and, at the 30-dpf phase, still characterized by not awakening the behavior of greater cohesion among individuals (Buske and Gerlai, 2011). However, the stress experience seems to have intensified cohesion behavior. The results obtained from group cohesion show that handling stress and social stress could trigger significant behavioral changes in young zebrafish. Some studies report that cohesive behavior represents an essential antipredator strategy (Herbert-Read et al., 2017; Ioannou et al., 2017) in addition to being considered a behavioral response that expresses the state of anxiety in zebrafish (Hamilton et al., 2017). Although we observed that handling led to increased cohesion in 30 dpf fish, the social stress used in the present study has exacerbated the response (Figure 1). Thus, it seems that recurrent disturbances in early development can modify the age pattern of shoal formation and cohesion response, and some stressors are more reliable than others in changing this response and provoking premature cohesive behavior.

After social stress exposure, zebrafish were held to grow up to 60 dpf, a stage of development in which morphological structures are already formed as an adult fish, but sexual maturity was not reached (Kimmel et al., 1995; Parichy et al., 2009). At this stage, fish did not present locomotion alterations due to early social stress, such as increased anxiety-like behavior or weakness. Thus, we showed that stress caused in the development interval between 20 and 30 dpf did not cause changes in motor patterns. Our results agree with those by Fontana et al. (2020) that used other stressors (water change, shallow water, and overcrowding) applied over 3 days at ~40 dpf. These authors found no effects of stress on locomotor and anxiety-related responses.

However, when considering the effects of early stress on zebrafish using the mirror test, a protocol that accesses fish motivation to approach and display aggressive signals toward its image, we observed differences in fish behavior: a decreased number of attacks against the image and increased distance kept from the interaction zone. These results could be interpreted as reduced motivation for social interaction, a behavioral feature that is characteristic of the zebrafish, a species considered highly social (Silva et al., 2019). The social avoidance response could be discussed in terms of the memory of the stressful situation experienced earlier, but there is no evidence supporting the idea that zebrafish retains memory for such a long time (Blank et al., 2009; Hieu et al., 2020). The formation of the adult aggressor–juvenile pairs was carried out randomly each day, thus avoiding that possible habituation among the individuals could reduce stress, once there was recognition among the individuals (Madeira and Oliveira, 2017).

A series of studies is dedicated to understanding how adverse childhood experiences (ACE) can influence individuals in adulthood, demonstrating that people who suffered any abuse in childhood are more likely to present destructive behaviors and more prone to drug addiction (Chapman et al., 2004; Herzog and Schmahl, 2018; Felitti et al., 2019). In rodents, attempts to simulate ACEs or early life stress are usually related to mother–offspring separation for a while, thus emulating behaviors of neglect or abandonment of young, and resulting in behavioral disorders in adulthood (Huot et al., 2001; Wei et al., 2010). However, using zebrafish as an animal model, the parental care approach could not be replicated (Spence et al., 2008; Tamilselvan and Sloman, 2017), but as a high need for sociability characterizes zebrafish, exhibiting typical responses of a state of stress when in social isolation, solitariness would be an alternative approach to abandonment observed in rodents (Fulcher et al., 2017; Shams et al., 2018).

In the present study, we show that early life stress caused by social threats and mistreatment caused behavioral changes related to higher cohesion within the conspecific group and social avoidance of unknown conspecifics without leading to anxiety and locomotor deficits. This is the first study to use zebrafish as a model of social stress during early development, and the findings suggest it could be a first step toward establishing a model for child trauma using zebrafish. This study opens a new path to understanding how juvenile trauma affects the brain and behavior, thus being able to contribute significantly to the understanding of this correlation between adverse experiences in childhood and detrimental behaviors in adulthood. More than that, we reinforce the reliability of the zebrafish as a sensitive tool for studies on the effects of early mistreatment and its consequences later in life.

## Data Availability Statement

The raw data supporting the conclusions of this article will be made available by the authors, without undue reservation.

## Ethics Statement

The animal study was reviewed and approved by Ethics Committee for Animal Use (CEUA-UFRN).

## Author Contributions

FM and IP carried out the experiment. FM and AL wrote the manuscript. FM conceived the original idea. AL supervised the project. All authors contributed to the article and approved the submitted version.

## Conflict of Interest

The authors declare that the research was conducted in the absence of any commercial or financial relationships that could be construed as a potential conflict of interest.
